# Co-creating the COMMUNICATE toolkit to support the communication of physical activity messages with adolescents in schools

**DOI:** 10.1186/s12966-025-01822-8

**Published:** 2025-11-11

**Authors:** Caera L. Grady, Elaine Murtagh, Maïté Verloigne, Kathleen McNally, Enrique García Bengoechea, Kwok Ng, Catherine B. Woods

**Affiliations:** 1 Physical Activity for Health Research Centre, Health Research Institute, University of Limerick, Limerick, Ireland; 2 Department of Physical Education and Sport Sciences, Faculty of Education and Health Sciences, University of Limerick, Limerick, Ireland; 3 Department of Public Health and Primary Care, Faculty of Medicine and Health Sciences, Ghent University, Ghent, Belgium; 4 Faculty of Education, University of Turku, Rauma, Finland; 5 Institute of Sports Science and Innovation, Lithuanian Sports University, Kaunas, Lithuania

**Keywords:** participatory research, whole-school program, peer-led, intervention, stakeholders, campaign, messages, health promotion

## Abstract

**Background:**

Communication campaigns within multi-component school-based interventions could improve knowledge and awareness about physical activity (PA) behavior. Guidance to implement such communication campaigns is lacking. This paper presents the co-creation and evaluation processes that led to the development of the COMMUNICATE toolkit, which supports implementers to communicate PA messages.

**Methods:**

Students and teachers from secondary schools enrolled in the Active School Flag (ASF) program were invited to participate. To provide a nuanced perspective on the communication of PA, ASF program implementers (i.e., coordinating teacher and adolescent peer leaders) and receivers (i.e., staff and students not involved in ASF delivery), together known as co-creators, engaged in three rounds of co-creation workshops to share ideas, provide feedback, and refine the toolkit. Workshop data were collected via activity recording sheets; written raw materials were photographed and later transcribed verbatim to generate a dataset. Inductive thematic analysis was conducted to organize and describe the toolkit components. A multi-stakeholder research steering group (*n*=7) was established to design, facilitate, and evaluate the co-creation process. The toolkit was refined between rounds of workshops. Throughout the co-creation process, the facilitator reflected after each workshop to improve its’ participatory nature. After the final workshop, co-creators completed a process evaluation questionnaire. Additional consultations with experts were held to bridge the gap in expertise. A logic model was developed to understand the theory of change behind the toolkit.

**Results:**

Eight teachers and 38 students from four ASF schools participated in the co-creation workshops. All 14 aspects of the process evaluation were mainly positive (86.7-100%). Common reasons for negative responses included co-creators not engaging, too much moving around during workshops, teachers’ involvement, and working with strangers. The final version of the toolkit included resources for program implementers to i) raise awareness about PA and the program, ii) plan the promotion of PA, and iii) develop key communication skills.

**Conclusions:**

The COMMUNICATE toolkit, informed by multi-stakeholder voices, emphasizes a multi-level, multi-stakeholder approach to communicating PA messages with adolescents in schools. It provides tools and resources for program implementers to improve communication efforts. The COMMUNICATE toolkit could be adapted to other peer-led school-based programs.

**Supplementary Information:**

The online version contains supplementary material available at 10.1186/s12966-025-01822-8.

## Background

Globally, adolescents have insufficient physical activity (PA) levels to obtain optimal health benefits [1]. To tackle the adolescent physical inactivity challenge, researchers have focused on developing effective interventions in the school setting; however, school-based interventions have been largely ineffective at changing adolescents’ PA behavior [2]. This lack of effect is often due to poor implementation; thus, more work is needed to ensure the successful implementation of school-based interventions [3, 4]. Multi-component, multi-level interventions that adopt a whole-school approach and an assets-based perspective to PA promotion provide a useful conceptual foundation for the design, implementation, and evaluation of interventions [5, 6]. However, the intricate multi-component nature, combined with schools being a unique, complex, and adaptive sub-system within a broader social system involving other institutions with an educational and health remit, makes implementation challenging [7, 8].

Implementation challenges were identified in the Active School Flag (ASF) program in Irish secondary schools [9]. The ASF is a whole-school, multi-component, multi-level PA program that aims to make “more schools, more active, more often”. The program implementers include a school PA champion teacher (ASF coordinator) and a class of adolescent peer leaders (ASF class). The ASF coordinator, supported by school management and a staff team, facilitates the peer leaders to complete various intervention activities, including but not limited to, administering a whole-school survey (student voice), running whole-school PA events (opportunity), and running a ‘Did You Know?’ campaign (awareness raising) within the school. An implementation evaluation conducted by McHale et al. [9] identified poor or a lack of communication within the program and the need for more support to implement specific program components, such as the ‘Did You Know?’ campaign. The ‘Did You Know?’ campaign aims to raise awareness about the benefits of PA and opportunities to be physically active within the school and community. A series of program changes were made in response to the implementation strategies proposed by McHale et al. [9]. The guidelines for the ASF ‘Did You Know?’ campaign changed from raising awareness about the benefits of PA and opportunities to be physically active in the school and the community (2018–2022) to sharing messages from the ASF class, about upcoming events, and general information about PA (2022–2025). Upon further examination, it became clear that there was a lack of evidence-based practice when communicating PA messages within the program, and more support was needed for the ASF program implementers to establish a successful communications campaign [10].

A PA message is ‘educational or persuasive material that is relayed to an individual, or group of people, with the aim of ultimately increasing physical activity levels’ [11]. A scoping review summarized how PA messages have been communicated with adolescents to date [12]. This review highlighted that the messages are usually shared within the school setting, during school hours, multiple times per week. The types of messages were usually educational, motivational, or feedback-focused, and adolescents preferred positively framed, funny, empowering, and autonomy-supportive messages. The message content would usually focus on the benefits of regular PA, overcoming the barriers, or suggestions and tips for PA. Half of the studies found in this review included a theory-informed messaging intervention (e.g., social cognitive theory, self-determination theory, theory of planned behaviour, and the transtheoretical model of behavior change) [12]. The differences between the outcomes measured and the methods used to communicate PA messages across the studies highlighted challenges for comparison and evaluation. Thus, there is a need to standardize the communication of PA messages with adolescents in practice (i.e., embedding evidence-based practices within existing multi-component school-based programs) and research (i.e., the evaluation of PA messages, to allow comparison across interventions and programs) [12]. The PA messaging framework developed by Williamson et al. [13] and the clear overview of how PA messages are communicated with adolescents by Grady et al. [12] provide a starting point to develop clear guidance for schools when communicating PA messages with adolescents.

Peers play a crucial role in influencing adolescent behavior; thus, it is important to consider them as potential communicators of PA messages [14]. Peer leadership in PA interventions is a useful way to promote PA among adolescents, especially in the school setting [15]. Peer leaders of PA programs often receive training on PA content, communication, intervention content, practice delivery, content design, and receive ongoing support [15]. Upon further examination of four peer-led PA interventions that provided communication training for peer leaders, only one provided enough detail about the training session content to allow for replication [15–19]. For peer leaders to fulfil their role in contributing to a communications campaign within a multi-component program, there is a need to understand and provide sufficient training to support their communication skills. However, due to the limited detail of existing communication skills training in PA programs, further work is required to develop this type of training.

One of the key factors outlined by Grady et al. [12] in a scoping review of communicating PA messages with adolescents highlighted the need to give adolescents autonomy over the messages they receive. Therefore, when developing guidance and support for communicating PA messages, it is important to actively involve those on the receiving end of the guidance or PA message. This can be achieved by conducting research *with* young people rather than *to*,* on*,* about*,* or for* young people [20, 21]. Engaging stakeholders in the development and implementation of initiatives is central to participatory research [22]. The careful attention to power dynamics (i.e., the end-user actively participating in the research process) suggests that participatory research is ideal for use with adolescents, particularly in school settings [21]. Freire et al. [21] conducted a review of participatory methods and approaches for engaging children and adolescents in decision making. This review highlighted six stages that children and family co-researchers engaged in participatory approaches (i.e., ideating, creating, refining, implementing, evaluating, sharing) [21]. These co-researchers were more likely to be involved in the earlier stages of intervention development (i.e., ideating, creating, refining) as opposed to the later stages (i.e., implementing, evaluating, sharing). The terms co-creation, co-design, and co-production are often used interchangeably to describe specific participatory research methods [23]. In this study, we refer to co-creation as ‘collaborative public health intervention development by academics working alongside other stakeholders’ [24]. The stages of the co-creation process align with the early stages of a participatory approach [23, 24]. Therefore, to ensure adolescent involvement at all stages, this unique study adopted both co-creation and a participatory approach.

Despite the recent advances in communicating PA messages with adolescents, it appears that whole-school PA program implementers currently do not have the necessary guidance or support to run successful communications campaigns [10]. This study aimed to address this need by collaborating with ASF stakeholders to develop resources to support whole-school program (WSP) peer leaders and coordinating teachers in communicating PA messages and to ultimately complement the overall WSP delivery. To the best of the authors’ knowledge, this is the first attempt to co-create tools and resources to support the implementation of a communications campaign within a WSP with peer leaders and coordinating teachers, while involving adolescents in decision-making at all stages of the participatory approach. This study describes the co-creation process with adolescents and teachers in secondary schools (methods), the process evaluation of the co-creation approach (methods and results), and the outcome of the co-creation process (i.e., the COMMUNICATE toolkit) (results).

## Methods

An iterative process of planning, conducting, reflecting, evaluating, and revising the design of the toolkit was followed to ensure a systematic approach to co-creation [24]. This study took place during the 2023–2024 academic year. The Consolidated criteria for reporting qualitative research checklist and checklist for reporting intervention co-creation were used (Additional files 1 and 2) [24, 25]. Ethical approval for this study was granted by the University of Limerick Faculty of Education and Health Sciences Research Ethics Committee [2023_04_08_EHS]. All participants provided written informed consent, or parental consent and child assent, before participating in the study.

### Planning

During the planning stage, there were two key principles to consider: framing the aim of the study and sampling. The PRODUCES framework was used to define the aim of the study (Table 1) [24].

**Table 1:** Co-creation PRODUCES framework

PRODUCES element	Defined as…
Problem	Communicating PA messages with adolescents
Objective	To design a set of resources to support/facilitate students/teachers
Design	Participatory action research
Users	Whole-school PA program implementers (peer leaders and coordinating teachers)
Co-creators	Secondary school students and teachers (ASF program implementers and receivers) and co-researchers (academic researchers, policy makers, adolescents, teachers, school management)
Evaluation	Process evaluation of the co-creation process
Scalability	Generalizable model (The generalizable model is to develop in collaboration with a sample of stakeholders (the co-researchers) and representative end-users of a larger population (the co-creators), a tailored intervention that can be scaled and implemented in a larger group (e.g., All ASF schools or schools with a similar WSP) (24))

#### Sampling

Balancing a representative sample of end users so the solution can be used by that group or scaled to a population level, as well as ensuring there is representation of all necessary expertise from relevant stakeholder groups, is challenging [24]. Therefore, due to the busy school environment and the available time and resources for this study, the core research team (CG, EM, CW) decided that two levels of participants, the co-creators (e.g., ASF program implementers and receivers) and co-researchers (i.e., various stakeholders with relevant expertise), were required.

**Fig. 1:**
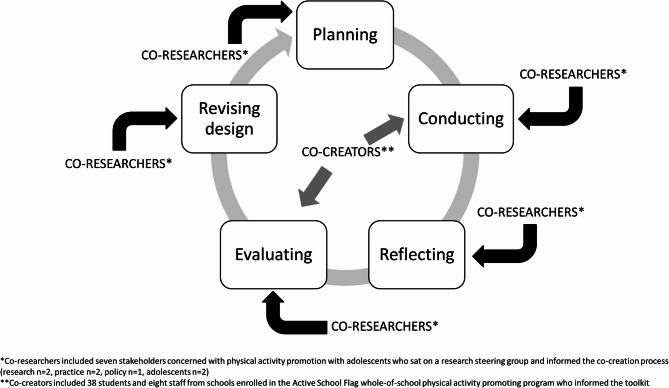
Stakeholders involved in the iterative co-creation process

The co-researchers were a purposeful sample of key stakeholders invited, from existing connections within the core research team, to join a research steering group during the planning stages of the study. The purpose of this research steering group was to make key decisions and inform the co-creation process. Co-researchers included seven individuals (71% female) representing policy (*n* = 1), practice (*n* = 2), research (*n* = 2), and adolescents (*n* = 2). These individuals included the first author (CG- a female PhD student researcher experienced with qualitative research), the third author (MV- a female international research collaborator with expertise in participatory research methods), one female policy maker from a government agency, two adolescent peer leaders (one male, one female), one female coordinating teacher, and one male school leader from an ASF secondary school. The co-researchers on the research steering group were separate individuals from the co-creators of the toolkit. The co-researchers were involved in the ideating, evaluating, and sharing stages, whereas the co-creators were involved in the creating and refining stages of the research process [21]. Figure 1 shows the different stakeholder involvement throughout the co-creation process.

Convenience and opportunistic sampling were used to recruit co-creators. A pre-existing relationship between the University of Limerick research team and the ASF school coordinators was utilised. CG contacted the ASF coordinator in six schools (two mixed-gender, two female-only, two male-only) from a mix of rural and urban areas, with more than three years of experience with ASF, to participate in a series of co-creation workshops. CG explained the study nature and requirements to the ASF coordinator. Once interest was expressed, a follow-up formal invitation was sent to school management. Each school was asked to recruit at random a small sample of staff (2–5 mixed between those involved and not involved in ASF delivery) and students (5–10 mixed between ASF peer leaders, non-peer leaders, junior, and genders to ensure a nuanced perspective) to engage in all workshops. The ASF peer leaders are in the fourth year of secondary school, called ‘Transition Year’ in Ireland, typically aged 15–16 years. Transition Year is a unique, optional, social and personal developmental non-academic ‘gap’ year between the junior (approximately 12–15 years) and senior (approximately 16–19 years) cycles in the Irish secondary school system [26]. In addition, parents (about 2–5) and management (about 1–2) from each school were invited to the final full-day workshop at the University of Limerick.

### Conducting

#### Manifesting ownership

In their first session, both co-researchers and co-creators were introduced to the main researcher (CG) (i.e., role, reasons for, and interest in the topic), given a brief introduction to the topic, and an overview of the current evidence base about the communication of PA messages with adolescents to facilitate their decision-making. Ownership differed for co-researchers and co-creators due to their roles. Firstly, co-researchers collaboratively drafted and agreed upon a statement of the research steering group’s rights and responsibilities. Furthermore, a shared folder was generated to ensure co-researchers had access to project materials.

Co-creators were informed about their roles, responsibilities, and rights as co-creators. Additionally, the co-creators were informed about the other schools involved in the process, the time required, the method of synthesizing their feedback (i.e., all schools’ feedback combined by the research team), and integrating their feedback into the toolkit after each round of workshops.

#### Defining the procedure

The co-researchers (members of the research steering group) had several roles and responsibilities, including making decisions about the design of the co-creation process, collecting, and interpreting the data. Seven meetings were held at the partnering secondary school between November 2023 and May 2024 at which decisions were made regarding the co-creation process.

Three rounds of co-creation workshops were run with four different schools between January and May 2024 (*N* = 11). The first two rounds were held at each respective school (approximately 1h each) and the final round was held at the University of Limerick as a full-day workshop (approximately 4h). All workshops took place during the normal school day. The ideas generated and feedback provided from each round of workshops were analyzed to establish a new version of the COMMUNICATE toolkit. All workshops in the individual schools were facilitated by CG and the final workshop was facilitated by the co-researchers and University of Limerick researchers.

A variety of commonly used participatory methods and activities for adolescents were used at each workshop to facilitate discussion, idea generation, and action among co-creators (Table 2) [21]. At the start of each workshop, the facilitator explained the study purpose, the focus for the workshop, reflected on the previous workshop(s) (if applicable), and performed an icebreaker. The co-creators engaged in independent thinking, group discussions, and recording of ideas generated and points discussed at each table on the activity sheets and post-it notes provided. All materials were photographed and later transcribed verbatim into data points. Further detail on the purpose and activities performed in each round of workshops is provided in Table 2.

**Table 2:** Purpose and description of the workshop activities in each round

Round	Location	Purpose	Activities ^a^	Participatory method or activity	Resources required (time, equipment
1	Each individual school (*N* = 4)	To introduce the co-creators to the study, outline their role as co-creators, familiarize the co-creators with the topic of communicating physical activity (PA) messages, and generate an initial version of the toolkit	Activity 1 (small group mapping): How is PA communicated in your school? – map out Who? What? Where? When? How?	Brainstorming Mapping	Average time: 69-minutes Equipment: Sticky notes, Pens, markers, flip chart paper
			Activity 2 (individual task): In your opinion what are the do's and don’ts when communicating PA messages with people your age?	Writing	
			Activity 3 (work in pairs): What are the challenges with communicating PA messages with people your age?	Discussion Writing	
			Activity 4 (small group idea generation): What resources (training, skills, tools) are needed to improve the communication of PA in your school?	Discussion Writing	
2	Each individual school (*N* = 4)	To review the prototyped version one of the toolkit and generate new ideas through two activities	Activity 1 (Small group idea generation): Review the thematic headings generated from round one and suggest physical resources that could be put in the toolkit to help the ASF team communicate PA messages	Brainstorming	Average time: 60-minutes Equipment: Sticky notes, Pens, markers, flip chart paper, prototyped toolkit
			Activity 2 (Ranking and reviewing prototypes): What are the ‘glows’ - areas that you like and think no changes are needed - and ‘grows’ - areas that need improvement or removal?- discuss and write down your thoughts – Use the sticky tabs provided to place green for glows and pink for grows and write your thoughts and discussions on the sheet provided	Using prototypes Ranking Discussion Writing Reflecting	
3	University of Limerick (*n* = 2) & individual schools (*n* = 2)	To address areas not already saturated with feedback for example, the language used, presentation, and the logistics of using the toolkit	Activity 1 (Review and critique): co-creators reviewed each aspect of the updated version of the toolkit in detail and provided feedback on the prototype.	Reflecting Discussion Writing	Full day workshop: 4h Individual schools: 45-minutes Equipment: Sticky notes, Pens, markers, flip chart paper, prototyped toolkit, round tables
			Activity 2 (Voting): co-creators voted on the presentation or format of each aspect of the toolkit.	Voting	
			Activity 3 (Ranking): Co-creators ranked their top 10 do’s and don’ts when communicating PA with adolescents (as identified in workshop 1).	Ranking	
			Activity 4 (Discussion): co-creators discussed the logistical elements to be considered with implementing and evaluating the toolkit.	Discussion	
			Activity 5 (Drawing/creating posters): co-creators worked together to create a poster that best represents the toolkit.	Drawing/creating posters	
			Activity 6 (mapping): Co-creators mapped the challenges identified in workshop one to aspects of the toolkit which is a strategy/solution to overcome the problem.	Mapping	
4	University of Limerick & online	Additional individual consultations were conducted to get advice on the design, implementation, and evaluation of the COMMUNICATE toolkit for the school setting from experts in marketing and communications, WSP^2^ development, equality, diversity, and inclusion, and graphic design		Discussion	The toolkit designs and plans

^a^Both staff and students engaged in the same activities during the workshops

Development (i.e., first version) and revision (i.e., following versions) of the toolkit were completed by CG, who was informed by suggestions from co-creators and literature on PA messaging, social marketing, and communication using an online graphic design platform [12, 13].

After the final round of workshops, additional consultations were held with experts from marketing and communications, WSP development, equality, diversity, and inclusion, and graphic design to complement the existing expertise of co-creators and co-researchers.

### Reflecting and Evaluating (the co-creation process)

A researcher reflection form and summary of each activity during the workshop were completed by CG immediately after the workshop to identify areas for self- and group evaluation [27]. The researcher reflection form consisted of 10 open-ended questions (e.g., “What went well?”, “What can be improved in the group process?”) followed by a table with 19 statements on a 5-point Likert scale. The reflection was completed after each workshop to allow the facilitator to openly reflect on the positive and negative aspects and ultimately improve the proceeding workshops. The researcher reflection forms were referred to during meetings with co-authors and the co-researchers, which provided an opportunity for CG to discuss any challenges and make decisions about future directions. The design and planning of the next workshop considered the reflection feedback to ensure a positive and holistic co-creation experience for all. For example, after the first workshop, the facilitator reduced the number of different activities and allowed more time for each, as the reflection after the first round of workshops highlighted the pressure to complete all activities within the allotted time.

In addition, a co-creator process evaluation questionnaire was administered at the end of the final workshop to evaluate the co-creation process [28]. Co-creators reflected on the process and responded to 15 statements on a 5-point Likert scale followed by two open-ended questions asking co-creators about what factors “facilitated” and “hindered” their experience during these workshops.

#### Sharing (the process and the findings)

The co-researchers conducted a series of dissemination activities to share the knowledge generated throughout the co-creation process. This included a conference presentation, the current scientific journal article, and a good practice example case study for a national ‘Young Voices in Decision Making’ government agency website. Three of the co-researchers (CG, coordinating teacher, policymaker) presented the co-creation process and the resulting toolkit at a local conference for researchers and knowledge-users in physical education, physical activity, and youth sport. The two academic co-researchers (and co-authors CG, MV) were responsible for writing the current article to disseminate the process and findings for researchers. Along with the first author, the two adolescent co-researchers co-authored a good practice example case study outlining the process of involving young people in decision-making, which is aimed at professionals and practitioners.

### Revising design (Data analysis)

All suggested changes for the toolkit from each school were documented by CG after each workshop. At the end of each round, all feedback was combined for analysis. After each workshop, a summary of each activity was generated by CG, incorporating any notes taken throughout the session. Data were processed whereby written raw materials from the workshop (e.g., post-it notes, brainstorms, etc.) were transcribed verbatim into a spreadsheet. Raw data from each school were stored securely for traceability purposes. The development and refinement of the toolkit took place between each round of workshops by CG, after analyzing and discussing the co-creators’ suggestions with the co-researchers.

After the first round of workshops, the suggestions for the toolkit from all schools were combined to generate the dataset. An inductive thematic analysis was conducted to minimally organize and describe the data in great detail [29]. The updated six steps to thematic analysis as outlined by Braun and Clarke [29] were followed: data familiarization and writing familiarization notes; systematic data coding; generating initial themes from coded and collated data; developing and reviewing themes; refining, defining, and naming themes; and writing the report.

To ensure a nuanced perspective of the data, CG worked with the co-researchers (who had varied experience and expertise) throughout the analysis process. CG was involved at all stages of data collection and analysis, which facilitated familiarization with the data. CG read the data and workshop summaries and wrote notes about ideas to explore and reactions to unpick. CG had good knowledge of the existing literature and practices for communicating PA messages and used this throughout the coding process to recognize patterns within the data [10, 12]. CG systematically coded the data, whereby each suggestion from the co-creators was given a code. To generate initial themes, CG worked with three co-researchers (two adolescents, one coordinating teacher) to minimally organize the data, develop, and review the themes. This involved an iterative process of grouping similar codes into categories and labeling each category. The resulting themes and subthemes were presented to all co-researchers and co-authors who acted as ‘critical friends’ in the final stages of refining and defining the themes [30].

The identified themes and subthemes formed version one of the COMMUNICATE Toolkit. This was presented at the second round of workshops and was the focus of the co-creators’ feedback. Thereafter, data from the remaining workshops were analyzed similarly, whereby each suggestion was coded, categorized with similar codes, and any deviant cases were reviewed individually. After the addition of new codes and updated feedback from participants, the themes and subthemes were revised to ensure they reflected the co-creators’ feedback. Each updated version of the toolkit was presented to the research steering group to identify activities and probing questions for the next round of workshops.

The development (i.e., version one) and refinement (i.e., following versions) of the toolkit was conducted between workshops. This study had a short timeframe (as it was conducted within the school setting), therefore, CG relied on her knowledge of the PA messaging literature from a recently conducted scoping review, which included similar WSPs with communications campaigns to ensure the toolkit was ready for the following workshops [12]. In addition, grey literature was searched for youth peer leadership training resources for communication skills and social marketing or communications campaign strategies and resources. For example, the PA messaging framework by Williamson et al. (2021) and existing PA messages developed for children and adolescents by Murtagh et al. (2024) were some of the resources adapted and embedded within the toolkit [13, 31].

The co-creators’ process evaluation Likert scale data were recoded into negative (0–1), neutral (2), and positive (3–4) responses for reporting purposes. Scale responses were analyzed descriptively and presented as percentages. Open-ended responses were analyzed separately, whereby responses to factors “hindering” or “facilitating” the session were analyzed using inductive thematic analysis following the same steps as previously outlined.

Once the co-creation process ended and feedback from the additional consultations were considered, the University of Limerick research team worked to develop a logic model to describe the theory of change of the COMMUNICATE toolkit.

## Results

The final sample of co-creators included eight staff and 58 students from four schools within the county of Dublin. This included two single-gender girls-only urban schools, one single-gender boys-only urban school, and one mixed-gender, rural school. Two schools declined to participate due to insufficient capacity to facilitate (i.e., a mixed-gender, rural school) and discontinuation of the ASF program (i.e., a single-gender boys-only, urban school). Within the participating schools, members of school management indicated it was not feasible to commit to a full day due to the busy time of the year. The coordinators in each school indicated that recruiting students at that time of the year was difficult, and getting parents to attend a full day would not be possible.

Almost 70% (*n* = 45) of co-creators responded to the process evaluation survey of which 91% were students. Only 35.6% attended all three workshops, 37.8% attended two of the workshops, and 26.7% attended one workshop.

In addition to the co-creators’ experiences throughout the process, this section also outlines the components, themes, and practical considerations that formed the final version of the COMMUNICATE toolkit. The additional files provided with this paper also showcase examples of the co-creators’ outputs from each of the workshops (Additional file 3), the visual changes to the COMMUNICATE toolkit model (Additional file 4), and a detailed overview of the changes made to the toolkit between each round of workshops (Additional file 5).

### Process evaluation

Four teachers and 41 students responded to the process evaluation survey. Findings indicated that co-creators mostly had a positive experience throughout the process. Five aspects of the process received a 100% positive response, and the remaining nine ranged between 86.7 and 97.8% positive. Negative responses were reported for seven aspects, ranging from 2.3 to 8.9% of responses. All teachers reported positive responses to all aspects of the workshops. The only barrier reported by teachers included students missing a workshop due to other commitments. The teachers reported the following factors that helped the workshops, including the facilitators, interactive nature, rotation of the groups, and collaborative working. Table 3 below shows a breakdown of the co-creators’ responses.

**Table 3:** Descriptive statistics of the co-creators’ process evaluation survey

	Response type (*N* = 45)		
*To what extent were the following aspects present…*	Negative n (%)	Neutral n (%)	Positive n (%)
Openness to new ideas			45 (100)
Exchange of useful information			44 (100)
Equal level of involvement	4 (8.9)	2 (4.4)	39 (86.7)
Climate of trust and openness		2 (4.4)	44 (97.8)
Positive atmosphere			44 (100)
Generation of new insight			44 (100)
Experience of joy	2 (4.5)	1 (2.3)	41 (93.3)
Effective leadership	1 (2.3)		43 (97.7)
Clear collective mission		4 (9.1)	40 (90.9)
Equal influences over decisions	3 (6.4)		41 (93.2)
Respectful interactions	1 (2.2)		44 (97.8)
Effective decision making	1 (2.2)		44 (97.8)
Satisfaction with progress			45 (100)
Use of understandable language	2 (4.4)	1 (2.2)	42 (93.3)

The co-creators’ open-ended responses, themed under the factors influencing their positive or negative experience during the co-creation workshops, are presented in Table 4. Despite independent analysis, there was an overlap between the positive and negative factors. It was clear from the responses that some co-creators had different experiences.

**Table 4:** Co-creators perceived factors influencing a positive or negative experience with the co-creation workshops

Positive experiences (N = 35)	Negative experiences (N = 37)
The respectful and open interactions between the co-creators created a positive environment (n = 9)	Co-creators found that there was a lack of engagement from some co-creators (n = 10)
Co-creators enjoyed the format and interactive nature of the workshops (n = 4)	Format and design of the workshop (e.g., too much moving around) (n = 9)
Co-creators felt the workshop facilitators were clear and well-prepared (n = 6)	Co-creators found some lack of clear instruction from facilitators (n = 5)
Co-creators felt their voice was valued and ideas were heard (n = 5)	
Co-creators enjoyed the collaboration of different schools and age groups (n = 7)	Co-creators did not enjoy working with people they did not know (n = 4)
Co-creators enjoyed sharing knowledge and generating new ideas (n = 4)	
	Co-creators did not enjoy teacher involvement (n = 2)
	No barriers (n = 7)

### Components and themes in the final version of the COMMUNICATE toolkit

The COMMUNICATE toolkit was refined from four themes (48 toolkit suggestions) after round one to three themes (87 toolkit suggestions) after round two, to finally three themes, 19 toolkit components, and one overarching theme of inclusivity and diversity. Table 5 outlines the various components of the final co-created COMMUNICATE toolkit.

**Table 5:** The COMMUNICATE toolkit themes and components

Theme		Component	Description
Theme 1: More emphasis on the whole-school program (WSP)	***Inclusivity and*** *** Diversity***	Introduction video for peer leaders	An introductory video for the class of peer leaders at the start of the school year to provide an overview of their role and the WSP.
		WSP themed 30-second game	Icebreaker or team bonding game for multiple players (teams of 2). Player 1 draws a card and has 30-seconds to describe all five words, player 2 tries to guess as many of the words on the card as possible. Rules: Player 1 is not allowed to say any of the words on the card. The person describing or guessing changes every round. The number of correct guesses is the number of places the team moves on the board. First team to get to the finish mark wins the game.
		Raise awareness about the WSP (e.g., sharing survey data with the rest of the school)	A series of topics to be covered when raising awareness: - Introduction the WSP - Recap on previous years activities - Celebrate hard work of previous peer leaders & official handover to new peer leaders - Share key whole-school survey findings & action plan - Encourage engagement with WSP - Others
		Create active campaign videos	Guidance for creating engaging, interactive videos.
		Identify key people to get the message across	Tips for identifying potential collaborators
Theme 2: Planning the promotion of physical activity (PA)		Steps for planning PA content	Step 1- Review previous efforts for communicating PA messages: Review all relevant channels that were used for communicating PA messages- social media, presentations, website, apps, posters, in-person announcements, emails- Identify the glows and grows
			Step 2- Class brainstorming activities to generate ideas for PA content: A series of questions to discuss in class: - How do we want our WSP to be viewed by others (i.e., social media, website, posters, etc.)? - What kind of PA topics do we want to communicate this year? - What times of the year/week should we communicate our messages? - How can we make our content more engaging? - Who else can we involve to communicate the message (i.e., collaborators identified in theme 1)? - How can we get teachers on board with the WSP throughout the year?
			Step 3- Meet with school social media manager or review social media policy Peer leaders arrange to meet with the staff member responsible for social media in the school. Discuss the schedule for posting, the expectations of each other, and the school’s policy for social media. Peer leaders should identify a list of questions prior to this meeting and clear them with the WSP coordinator (e.g., frequency and availability for posting, format of sending and receiving content to be posted, need for a template or checklist to ensure all relevant information is included such as, a caption, people to tag and music for the post).
			Step 4- Content planning template Use results from the brainstorming activity to fill out the template which clearly outlines all the information for each piece of planned content (online or in-person). This can be shared with the WSP coordinator, school leadership, and social media manager for approval before content is created to ensure transparency and opportunity to provide feedback.
		Tips to boost social media	Video for peer leaders highlighting things to consider when posting on social media
		PA message tools	Option 1: Adapted PA messaging framework to develop your own PA messages Option 2: Existing scientifically tested and valid PA messages
		General tips for promoting PA	Do’s and don’ts for communicating PA messages with adolescents
Theme 3: Develop communication skills		Identifying class skills needs	Conduct skills need assessment amongst the peer leaders through group discussion. Small group discussion within their peer leadership teams- identify the skills needed to carry out their roles and responsibilities. Class discussion: List all common and unique skills needed. Individual activity- write down one strength and one weakness in their skillset to work on this year. Coordinator decides based on assessment the order in which skills need to be developed.
		Skill development	Listening skills To help students understand the impact of the language they use, how people can interpret it differently to their meaning, and the importance of planning what you are going to say before you say it.
			Presenting and public speaking - Video of tips for presenting and public speaking - Challenge cards to practice public speaking- beginner, intermediate, and advanced challenges – peer leaders pick a card and have one week to complete the challenge (e.g., volunteer to make the next intercom announcement about the WSP)
			Problem-solving and decision-making - Discuss problem-solving steps - Scenario card activity- series of scenarios that the peer leaders may face in their roles. Peer leaders work in groups to review the scenario and identify a solution. The different scenarios and solutions are discussed with class.
			Teamwork - Icebreaker activities (e.g., 30-seconds, challenge cards and problem-solving scenario cards) - Identifying opportunities to collaborate (i.e., Sect. 1)

#### Theme 1: putting more emphasis on the existing whole-school program

Schools need to emphasize the importance of their WSP to make it more recognizable and create more interest amongst the school community. This can be enacted through awareness-raising. Firstly, the peer leaders need to be aware of their role and the influence they have on the school community. Co-creators suggested that the class of peer leaders should receive “proper introductions to Active School Flag and the activities they run or will be running- maybe with an introduction video” (Teacher, school 1). After the peer leaders are familiarized with the program, they perform a series of activities to raise awareness about the WSP, including (i) introducing the program to the school community for example, “an assembly dedicated to ASF to encourage engagement” (Student, school 3); (ii) reflecting on the progress from previous years; (iii) celebrating the hard work of the previous peer leaders and have an official handover of the implementation role such as, the “previous ASF team act as mentors or prefects throughout the year to help out the new team” (Student, school 1); (iv) presenting their school’s activity data (from survey data) and the resulting action plan for the year for example, “give background to ASF- presentation examples of past events” (Student, school 1); (v) informing the school community how they can get involved in the program for example, “get other year groups involved on social media” (Student, school 2); and (vi) raising awareness to the local community such as, “put ASF posters up around the local community” (Student, school 2).

Awareness raising should continue throughout the school year and be supplemented by creating active campaign videos to encourage more people to be more active, more often. Finally, to put more emphasis on the WSP, the peer leaders should seek to collaborate with key people to help get the message across, for example, “getting well-known people to promote the events” (Student, school 3).

#### Theme 2: planning the promotion of PA in the school

This section of the toolkit will help WSP implementers recognize the best practices when communicating PA messages, which involves planning what you will say in advance. The toolkit outlines (i) a series of “steps or actions” for the peer leaders to follow when planning their PA message content for social media or other platforms, (ii) tips to help peer leaders boost their social media practices as they requested “ideas on how to promote PA in the school (e.g., on social media- reals, vlogs, plogs)” (Student, school 3), (iii) tools for coordinating teachers to use with the peer leaders to create their own PA messages or pick from a list of pre-developed and tested PA promoting messages as teachers requested material like “resource cards for did you know campaign – a template for everything you need and sample websites to get information from” (Teacher, school 1), and (iv) general “ideas on how to promote PA in the school” for peer leaders to consider when communicating. The tools and resources presented in this part of the toolkit can also facilitate the activities in the first section of the toolkit (putting more emphasis on the WSP).

#### Theme 3: developing communication skills

The final area of the toolkit presents a series of activities and exercises for the WSP coordinating teachers to utilize with the peer leaders to improve their communication, teamwork, problem-solving, and decision-making skills and competencies. Activities and exercises included in this section include suggestions from co-creators to develop key communications skills including, (i) “listening skills activities” (Teacher, school 1), (ii) “presentation” and “public speaking” skills exercises (Students, school 1 and 2) such as, presenting “to smaller groups and slowly work up to classes, then bigger audiences- start with younger years then build up to seniors” (Student, school 4), (iii) “problem-solving” and decision-making scenarios (Teacher, school 3), and (iv) “teamwork” and developing relationships within and outside of the WSP implementation team (Student, school 4) for example, an “ASF 30-seconds game for team bonding” (Student, School 1).

#### Overarching theme of inclusivity and diversity

As evident in Table 5, inclusion and diversity underpin the entire toolkit. The co-creators were passionate about the importance of ensuring and emphasizing that ASF and PA, in general, are for everyone. The co-creators wanted the toolkit to provide ideas on how to engage the hard-to-reach people in activities “e.g. bring treats, make it non-competitive, suitable for friend groups” (Student, school 4), ideas for “inclusive games: boules, obstacle courses, tasks to complete around the walkway” (Teacher, school 1), emphasis on communicating “during and after the event, ask for feedback- how is it going, what would work better next time” (Student, school 4), and “emphasis should always be about having fun” (Student, school 4). The toolkit encourages a classroom discussion in each section to remind peer leaders that their actions and communication efforts should cater to inclusion and diversity. Furthermore, the toolkit provides suggestions on aspects to consider.

### Practical considerations for the COMMUNICATE toolkit

As previously outlined in Table 2, the final two rounds of the co-creation process focused on the design, format, presentation, and future of the COMMUNICATE toolkit. The co-creators suggested that the toolkit should contain (i) a teacher’s manual, (ii) a student slideshow, and (iii) a box of resources. It should be available in both digital and physical formats. It was suggested that the toolkit was a hybrid teacher-student resource for whole-school PA programme implementers to use. The co-creators designed a cover page that best represents the contents of the toolkit which was later created by a professional graphic designer (Additional file 6).

The additional expert consultations were conducted to build on the existing expertise of the co-creators and co-researchers, yielding valuable insights into the marketing and communications of the toolkit, embedding the toolkit into existing programs, and the graphic design of the toolkit. Feedback from these consultations addressed the future scale-up of the toolkit concerning, (i) the advertisement, promotion, and branding of the toolkit for relevant audiences, (ii) adaptation to different settings, including the language used, branding and feasibility of certain aspects of the toolkit in varying contexts, iv) the use of technology and online platforms to host the toolkit as well as record and monitor its use and engagement, and v) ultimately measuring its impact.

### Logic model of the COMMUNICATE toolkit

A logic model was developed by the co-authors to understand the theory of change behind the COMMUNICATE toolkit and to facilitate its evaluation. The co-creators and co-researchers suggested that this should be decided by researchers with the appropriate knowledge. Presented in Table 6, the logic model describes that the coordinating teacher uses the COMMUNICATE toolkit with the adolescent peer leaders of a whole-school PA program to improve their skills and competencies for communicating PA messages. The toolkit helps peer leaders to: (i) raise awareness about PA and ASF amongst themselves and the wider school community through encouraging value recognition for PA and the ongoing WSP, (ii) recognize best practices for communicating PA with peers through planning and understanding their school context, and (iii) increase capacity to communicate PA more efficiently and effectively by developing communication skills to improve their social media, public speaking, listening, team working, and problem-solving. We hypothesize that if the program implementers are provided with a set of resources to support the communication of PA messages within the school, this will enhance the PA knowledge, awareness, and eventually behavior of the wider school community.

**Table 6:** Logic model of the COMMUNICATE toolkit

*The COMMUNICATE T* *oolkit Logic Model* Aim: To provide whole-school program (WSP) implementers (i.e., teachers) with resources to increase adolescent peer leader implementers with skills and competencies for communicating physical activity (PA) messages through raising awareness, increasing capacity, and recognizing best practices.			
INPUTS	ACTIVITIES	OUTPUTS	OUTCOMES
-COMMUNICATE Toolkit induction online session -COMMUNICATE Toolkit -WSP coordinating teacher’s commitment to deliver the toolkit -Timetabled class with peer leaders -Training days for implementers of WSP (program specific) -WSP/PA social media page(s) -WSP coordinator informs peer leaders about the program, their roles, and responsibilities (i.e., in communicating PA) Students get: -Introduction to the WSP and understand the importance of roles - Class portfolio to document activities completed within the WSP.	(What the students do) **Value recognition** -Celebrate WSP efforts -Motivate and encourage people to engage with the WSP -Identify and engage relevant stakeholders **Planning** -Audit previous WSP communication efforts -Brainstorm target audience needs, desires and preferences for PA messages -Create PA messages -Collaborate with key stakeholders -Learn how to use social media efficiently and effectively **Skill Development: communication skills** -Use practical and challenging tasks to bridge competency gaps -Apply new knowledge to communicate PA more effectively and innovatively	(What students produce) -Document awareness-raising activities in portfolio -Active campaign video(s) shared on the school website, social media, and in classrooms -List of key stakeholders in the portfolio -Padlet of audit and brainstorming activities completed -Content planning templates complete -Bank of messages created and delivered -Record of collaboration(s) -Practical activities and tasks completed -Reflection on the communication of PA messages throughout the year in the portfolio	Short term -Improved communication self-efficacy for peer leaders (intrapersonal) -Improved team working skills among peer leaders (interpersonal) -Increased PA knowledge, awareness, and beliefs among students (intrapersonal) -Increased knowledge and awareness of the program within the school (organizational). -Increased PA motivation, self-efficacy, and social norms (intrapersonal). -Enhanced school-relatedness, school-satisfaction, sense of belonging, social connectedness, group environment, student participation with organized activities (organizational). Long Term -Enhanced PA attitudes and social norms (organizational). -Enhanced PA levels (organizational). -Increased knowledge and awareness of the program in the community (Community)
Assumptions: The school has a whole-school PA program that is peer-led, supported by teacher(s) and school management. The WSP has a ‘communications campaign’ component. If the WSP implementers are provided with a set of resources to support the communication of PA messages, the knowledge and awareness of PA and the WSP among the school community will improve.		External Factors: The COMMUNICATE toolkit is dependent on the normal day-to-day functioning of the school environment therefore, environmental factors that may result in school closures will impact its delivery.	

## Discussion

This study aimed to co-create a toolkit with WSP implementers and receivers to improve the communication of PA messages within the school. Students and teachers, both involved in the delivery of the ASF program and not involved in the delivery, engaged in the co-creation process involving the ideating, creating, refining, and implementing stages of a participatory approach. A smaller sample of school stakeholders representing adolescents, school leadership, teachers, and school policymakers was involved in the later stages of a participatory approach, including evaluating and sharing. Overall, the co-creators had a positive experience throughout the co-creation process. The COMMUNICATE toolkit provides resources for WSP implementers to help raise awareness about PA and the WSP, plan the promotion of PA, and develop communication skills. A theory of change was developed highlighting the potential of the COMMUNICATE toolkit to improve skills and competencies on the intra- and interpersonal levels for peer leaders and knowledge, and awareness at the intrapersonal and organizational levels.

Firstly, the COMMUNICATE toolkit emphasizes raising awareness about PA and the WSP, as raising awareness can be considered the first step in the behavior change process [32, 33]. Albert Bandura [34] stated that in order for a person to change their behavior, they first need to know how to perform the behavior, be motivated, and have confidence; therefore, if adolescents are aware of the program and what it involves, they would be more likely to engage with it.

Secondly, the toolkit recommends planning the promotion of PA as it is an integral part of health promotion intervention development [35]. The COMMUNICATE toolkit emphasizes engaging multiple stakeholders when planning and communicating PA messages. Similarly, a systems approach within the school setting aims to bring key stakeholders together to help them work collaboratively and clearly to address the complex challenge of PA promotion with adolescents [6].

Finally, the COMMUNICATE toolkit is aimed at WSP implementers, specifically coordinating teachers and adolescent peer leaders. The COMMUNICATE toolkit stresses the importance of developing peer leaders’ communication skills to carry out their role as communicators of PA messages. A systematic review of peer-led strategies outlined that only 44% (*N* = 18) of the included studies provided training for peer leaders, and a mere 22% provided training for peer leaders to develop communication skills [15]. Furthermore, the details of the communication skills training were not always well reported. However, Sebire et al. [16] (i.e., the Peer-Led physical Activity iNtervention for Adolescent Girls (PLAN-A) study) provided details about the included written and practical role play activities, building awareness of communication skills and practicing their use. This is similar to the activities provided in theme three of the COMMUNICATE toolkit.

Alternatively, standalone mass media campaigns such as the VERB or WIXX provide good practice examples of using social marketing to implement large-scale multi-media campaigns [36, 37]. However, the applicability of such campaigns to the school setting should be carefully considered. Such campaigns can be heavily resource-intensive; thus, the sustainability (i.e., continued delivery) of the intervention is questionable. The COMMUNICATE toolkit provides WSP implementers with resources to plan their own communications campaigns thus, the toolkit is adaptable to different contexts and has greater potential for sustainability.

On a similar note, the HEALTHY study includes a communications component that involved the use of branding, posters, messages, student peer communicators, events, and merchandise to reduce risk factors for diabetes [38]. The HEALTHY and COMMUNICATE communications interventions are similar in that they are developed and implemented with students and other school personnel. In contrast, the HEALTHY study focused on delivering five specific campaigns over five semesters, whereas the COMMUNICATE toolkit provides the tools and resources for repeated year-long communication campaigns. Furthermore, the student peer communicators in the HEALTHY study acted as channels for sharing the message as opposed to those in the COMMUNICATE toolkit (i.e., peer leaders are the implementers of the communications campaign).

The COMMUNICATE toolkit is largely underpinned by social cognitive theory, the socio-ecological model, and the precaution adoption process model [33, 34, 39]. For example, the COMMUNICATE toolkit works on multiple levels such as, the individual (peer leader), interpersonal (students), organizational (whole-school community), community, and public policy levels [39]. The objectives of communications campaigns usually focus on changing knowledge, attitudes, beliefs, motivation, and human interactions, which work towards achieving behavioral, social, or organizational objectives and the overall program’s goal [40]. Therefore, the COMMUNICATE toolkit targets these determinants of PA rather than expecting a direct behavior change. The COMMUNICATE toolkit operates whereby the WSP implementers are provided with the resources, tools, knowledge, skills, and thus, their capability to ultimately improve their self-efficacy to communicate PA messages [32, 34]. This aligns with findings from the review by McHale et al. [15] that stated the need to move beyond measuring PA outcomes for peer leaders and their peers towards the broader physical, social, and mental benefits of peer-led interventions. Peer leaders of PA interventions have the capacity to develop new skills in their roles, but more research on the long-term impact of being a peer leader of a PA intervention is needed to identify suitable long-term goals [9, 41, 42].

The theory of change (i.e., logic model) outlines several assumptions for the COMMUNICATE toolkit to work. This includes an existing WSP structure with peer leaders, program coordinators, and a communications campaign component. Nevertheless, as the COMMUNICATE toolkit is an intervention itself, implementation support will be required to understand and use the toolkit. However, the extent of this is yet to be determined. In the HEALTHY study communications campaign component, study personnel and staffing, marketing firm or social marketing expertise, and technical or production expertise for student-generated media activities were some examples of the support required for successful delivery of the campaigns [38].

Overall, co-creators generally had a positive experience in this study, which is in line with some other co-creation process evaluation studies with adolescents and school staff [43, 44]. In contrast, a scoping review that examined school staff experiences of engaging in a co-creation process highlighted that the barriers to engaging outweighed the benefits due to the substantial demands of their job [45]. However, this review notes that in the included studies, students were the primary population of interest and thus, their needs were prioritized over the staff which may explain the negative staff experiences [45]. This was not the case in this current study as the teachers’ insights and perspectives were as valuable as the students. Familiarization with the common pitfalls in engaging young people throughout the co-creation process, such as a lengthy timescale or the power sharing dynamics, was a success factor in ensuring the adolescent co-creators had a positive experience, that their voice was heard, and that the power was handed over to them during the workshops [21, 46, 47]. This study highlights that it is possible to give adolescents autonomy to make decisions about topics that affect them, and it can result in the successful co-creation of an intervention. The regular reflection practices by the facilitator was a key success factor in respecting the power balance and ensuring a meaningful co-creation process. A similar reflection process was followed by Maenhout et al. [27] who highlighted the importance of the researcher recognizing their own reflexivity. The researcher reflection forms became more positive over time as areas for improvement were identified, discussed with co-researchers, and subsequently addressed in between workshops to ensure a meaningful co-creation experience.

There are some lessons to be learned from this co-creation approach. Firstly, the nuanced experiences of the co-creators should be explored further in future research to determine if attendance at all or only some workshops impacts perceived experience. Next, the involvement of teachers was a factor that some students did not appreciate. However, from the researcher’s perspective, this helped provide a broad perspective of the school context, which was also noted by Maenhout et al. [27]. A clearer explanation from the facilitator of the value of each co-creator at the start of the co-creation process may have created a better dynamic. Finally, working with a mix of people, including some unfamiliar with each other, was another factor that some co-creators did not enjoy. This was unexpected as developing social connections with fellow youth advisors was previously identified as a benefit, albeit in older youth [48]. Similarly, a clearer explanation at the start about the process involving working with unfamiliar or new people may have provided an opportunity for the co-creators to express concerns in advance.

### Applications to research, practice, and policy

This study demonstrates a unique method of involving multiple stakeholders in each stage of a participatory approach, especially adolescents, through the use of two different stakeholder groups, co-creators and co-researchers. This is a feasible approach that could be adopted by researchers conducting participatory research within the school setting to maximize engagement. This toolkit provides a first attempt to support WSP implementers in communicating PA messages. Future directions for this research include the need to conduct testing on the toolkit to evaluate the process of implementing it in practice, its feasibility, and effectiveness. The toolkit described here could also be tested in other settings (e.g., in the community or sports clubs), or the findings could be adapted through further co-creation in different contexts.

For practitioners, this toolkit provides pragmatic tools and resources developed with school stakeholders that could be added to an existing WSP to improve the communication of PA messages with adolescents and support program implementers throughout the process. For policy, this toolkit has potential to improve existing WSP with minimal cost to implement. Furthermore, this toolkit could inform the first national strategy or framework for communicating PA with adolescents in schools.

### Strengths and limitations

The strengths of this study include the involvement of a multi-stakeholder research steering group, in particular, adolescents in the research and decision-making processes. One researcher facilitated all workshops and also analyzed the data, which helped with consistency and familiarization throughout the process. The active reflection practices between the workshops helped the researcher recognize, discuss, and overcome any strong feelings or views that were raised during the co-creation process.

Some limitations to consider in this study were the timeline constraints. The study was conducted within the school setting which is a very busy environment and has many other competing priorities. It was decided that the study would be conducted within one academic year to keep the topic fresh for co-creators and to overcome common barriers to co-creation such as the lengthy timescale involved [47]. This led to a lack of time to discuss a group contract with the co-creators and the systematic and thorough searching of materials for the toolkit.

As the workshops took place during the school day, which was deemed most appropriate by the co-researchers, only one facilitator was available for most workshops; nevertheless, all co-researchers were involved in the final workshop. Furthermore, there was only one researcher who completed the researcher reflection forms which limits the perspective to one point of view. Thus, future research would benefit from having more co-researchers involved during all workshops to ensure accuracy of recording and reflection at each workshop. The workshops took place during the school day, which was deemed most appropriate by the co-researchers, this required students to miss class time, and may have limited the involvement of students in examination years. Finally, if a student was absent from school on the day of the workshop or unable to attend at that particular time, they were not able to provide feedback until the next round of workshops. In future, if conducting multiple co-creation workshops with adolescents in the school setting, researchers should plan mechanisms to encourage continued participation of co-creators throughout. Finally, the process evaluation did not allow for in-depth discussions with co-creators about their positive and negative experiences during the co-creation workshops.

## Conclusions

This study described the development of, and the resulting, co-created COMMUNICATE toolkit which included practical tools and resources for WSP implementers to improve the communication of PA messages with adolescents. This toolkit was co-created with stakeholders from ASF secondary schools in Ireland but can be adapted to similar peer-led school-based programs. It is possible to involve adolescents in decision-making at all stages of a participatory approach. Furthermore, this study integrated and empowered the student voice from conceptualization through the development of an intradisciplinary research steering group. Although this toolkit considered some practical aspects relating to sustainability and scale-up, further research is needed to test the feasibility, effectiveness, and implementation of this toolkit within an existing WSP.

## Supplementary Information

Supplementary Material 1.

## Data Availability

The datasets used during this current study are available from the corresponding author on reasonable request.

## Abbreviations

PA: Physical Activity
ASF: Active School Flag
WSP: Whole-school program
PRODUCES: Problem, objectives, design, end-users, co-creators, evaluation, scalability
