# Supplementary material for: Co-creating the COMMUNICATE toolkit to support the communication of physical activity messages with adolescents in schools
Source: Int J Behav Nutr Phys Act. 2025 Nov 11;22:140. doi: 10.1186/s12966-025-01822-8 (PMC12607130; doi:10.1186/s12966-025-01822-8)
Supplement: Supplementary file 1 — Supplementary Material 1. [file 12966_2025_1822_MOESM1_ESM.docx]

**Additional files for manuscript titled “Co-creating the COMMUNICATE toolkit to support the communication of physical activity messages with adolescents in schools” in IJBNPA**

**Authors:** Grady, C. L., Murtagh, E., Verloigne, M., McNally, K., Bengoechea, E. G., Ng, K., Woods, C. B.

**Additional File 2- Checklist for reporting intervention co-creation**

| **Checklist item** | **Page number** |
| --- | --- |
| Use each element of the PRODUCES framework | 7-9 |
| Explain the criteria used for sampling | 9-10 |
| In what settings did sampling occur? | 9-10 |
| How many individuals engaged as co-creators? | 17 |
| Describe the co-creators | 9-10 |
| Explain the methods used to manifest ownership | 11 |
| What level of participation was there from the co-creators? | 12-13 |
| How was the overall aim presented? | 12-13, Table 2 |
| How was the purpose of each meeting presented? | 13, Table 2 |
| What were the rules and responsibilities of participation agreed upon? | 12 |
| In which areas did the co-creators require upskilling? | 12 |
| What previous evidence was reviewed, and how? | 12, 14-15 |
| If a prototype was developed, describe the prototype and the prototyping process | 12-16, Additional file 2,3,4 |
| Describe the frequency and duration of meetings | 12-13 |
| Give examples of interactive techniques or methods used | 12-13, Table 2 |
| Give examples of fieldwork techniques or methods used | 14-15, Table 2 |
| Give examples of how iteration occurred during the process | 9-13, Fig. 1 |
| Explain how co-creator satisfaction and contribution was evaluated. | 14 |
| Explain how the validity of the outcome and the process were evaluated. | 12-14 |
| Explain plans for formal testing of the effectiveness/scalability of the co-created outcome | 29 |
| Explain outcome of evaluation (if tested) | N/A |

**Additional file 3- Examples of the co-creators outputs from the activities conducted during co-creation workshops**


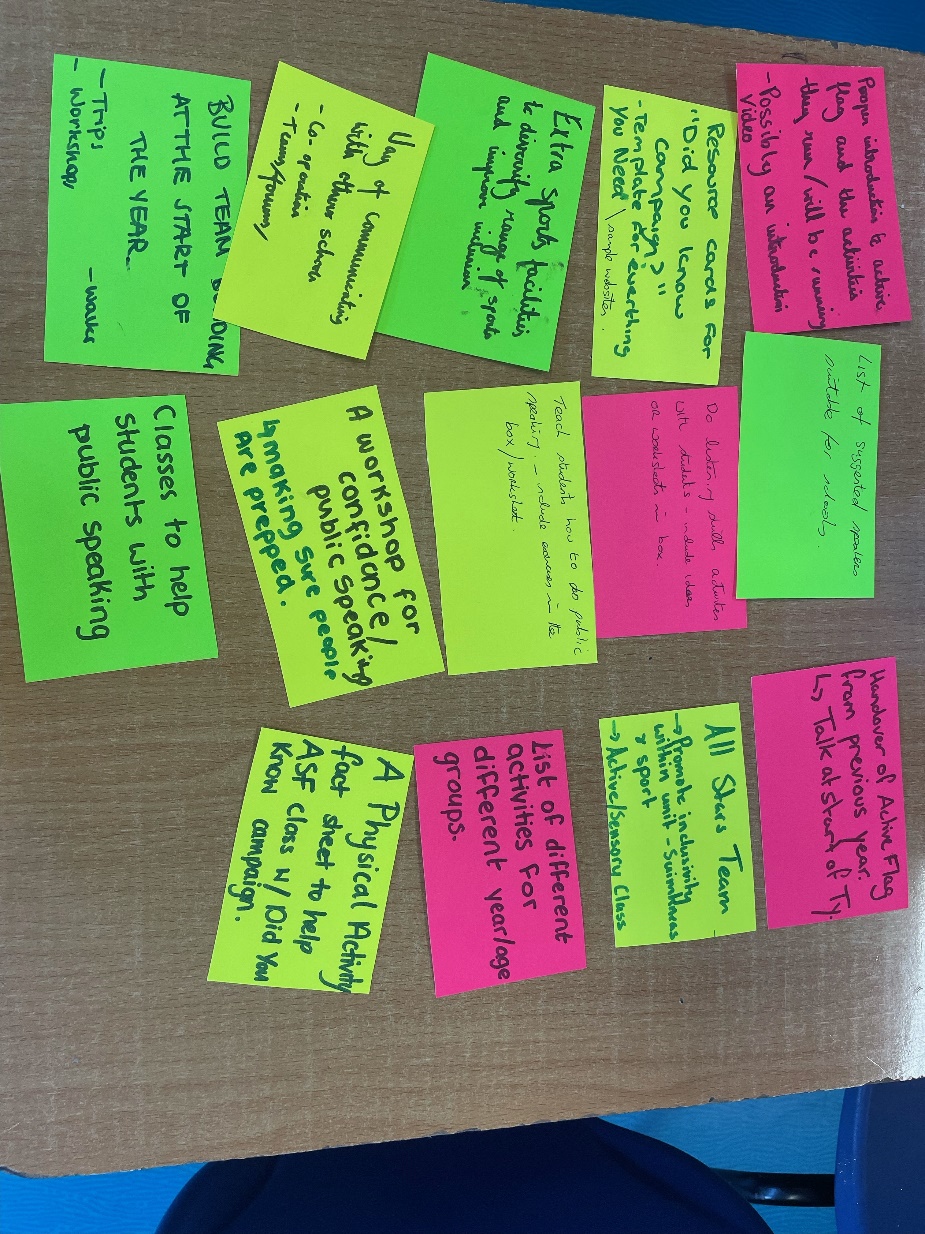


*Figure 2a. Example of co-creators suggestions for resources to put in the toolkit at workshop 1*


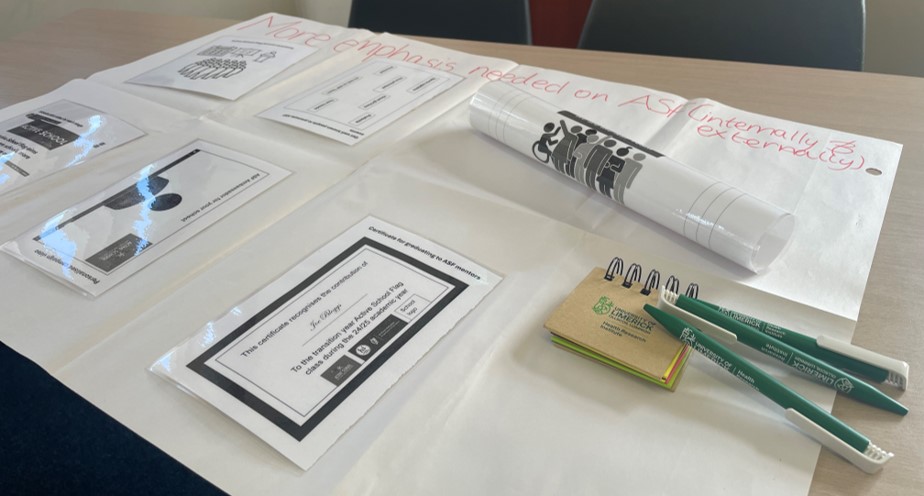


*Figure 2b. Example of the prototyped COMMUNICATE toolkit version 1 resources presented at workshop 2*


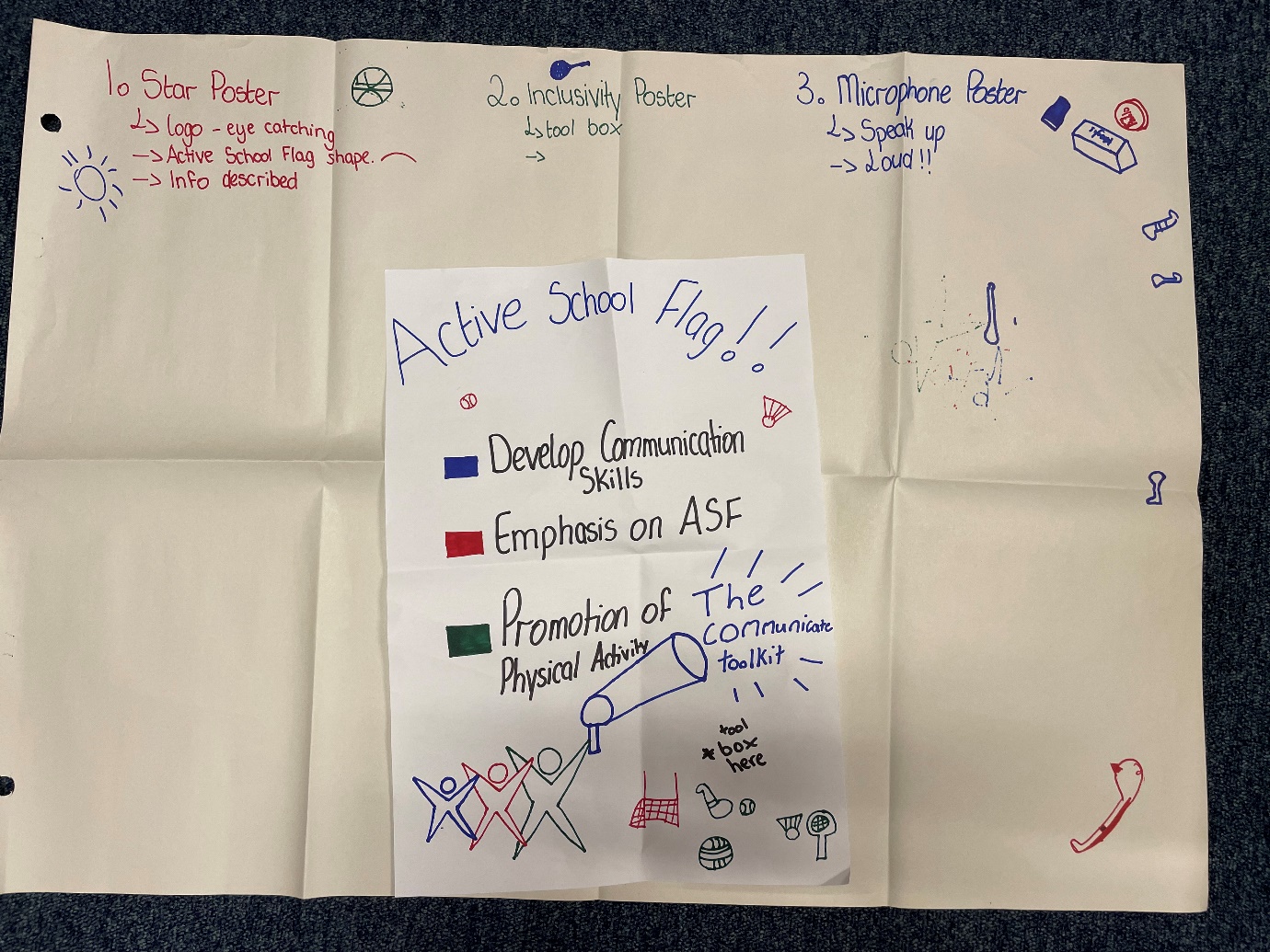


*Figure 2c. Example of the drawing activity (designing the cover page of the COMMUNICATE toolkit) at the final workshops*

**Additional File 4- Illustration of the COMMUNICATE Toolkit model changes throughout the co-creation process**


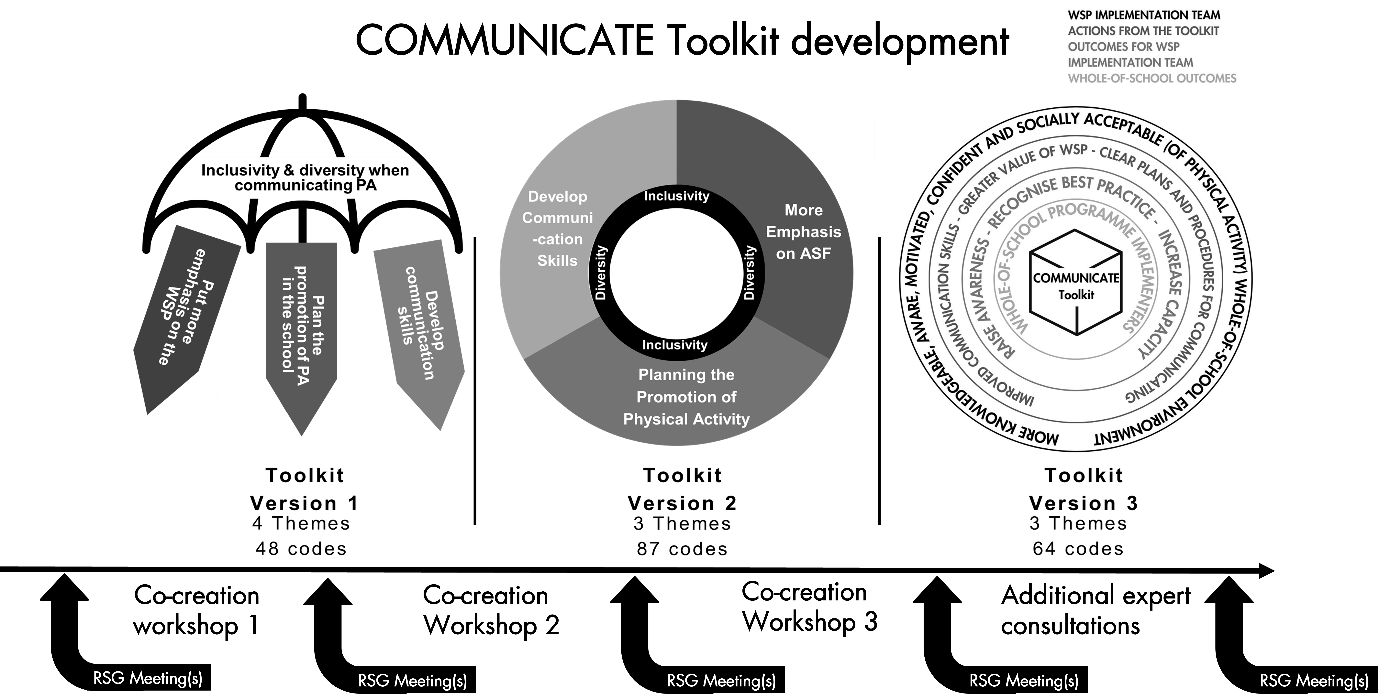


**Additional File 5- Overview of the changes in the suggested tools and resources throughout the co-creation process**

| **Thematic area** | **Content** | **Version** | | | **Explanation of changes at each round** |
| --- | --- | --- | --- | --- | --- |
|  |  | **1** | **2** | **3** |  |
| 1.0 More emphasis needed on the whole-school program | 1.1 Annual Active whole-of-school program Ceremony | ✓ | ✓ | ⭮ | 1. **Kept.** Added suggestions for how this may work within the school environment e.g. the time of year, the people involved, the information shared. 2. **Kept.** Suggested to allow schools flexibility to do what works for them 3. **Revision necessary.** One whole-of-school ceremony is unlikely to work. Rather a series of events take place, are documented and showcased to the rest of the school community through various platforms. |
|  | 1.2 Introduction to ASF videos | ✓ | ✓ | 🗶 | 1. **Kept.** Suggested that there should be one for the implementers and a different one for the rest of the school. Suggestions provided as to what should be included in these videos. 2. **Kept.** Suggestion to improve existing whole-of-school program introduction video and create different ones for each stage of the program. 3. **Removed.** Outside of the scope of this toolkit, should be strongly recommended to whole-of-school program developers to action as part of the program. |
|  | 1.3 Certificates for student implementers | ✓ | ✓ | ✓ | 1. **Kept.** Proposed questions to understand how these could be recognized in the outside world. 2. **Kept.** Template is good but should consider also a digital badge from whole-of-school program developers. 3. **Kept.** No further changes suggested. |
|  | 1.4 Handover baton | ✓ | ⭮ | 🗶 | 1. **Kept.** Provided suggestions for who should do the handover. 2. **Revised.** Suggested that it should be strongly recommended to whole-of-school program developers but not directly needed within the toolkit. 3. **Removed**. |
|  | 1.5 Campaign video | ✓ | ✓ | ⭮ | 1. **Kept.** Suggestions for potential ambassadors for their active school. 2. **Kept.** Suggested having one campaign video for advertising at national level and one at the individual school level. 3. **Revised.** Suggested that schools create their own active campaign videos internally. |
|  | 1.6 Collaborators | ✓ | ⭮ | ⭮ | 1. **Kept.** Suggestions for additional potential active school collaborators. 2. **Revised.** Moved to next section - Additional people added for within school and outside of school collaborations. 3. **Revised.** Suggested that this should be referenced for multiple aspects of the toolkit e.g. when putting more emphasis on the whole-school program, when planning the promotion, and when developing communication skills. |
|  | 1.7 Inclusivity | ⭮ | ✓ | ✓ | 1. **Revised.** Suggested that it was unclear having inclusivity as a standalone section, there was too much overlap. Suggested to try to integrate better with rest of toolkit by considering inclusivity in each section. 2. **Kept.** Suggested that having class or year group reps could help other year groups feel included rather than involvement being limited to the whole-school program implementers. 3. **Kept.** Create a checklist or poster to remind the whole-school program implementers to check if they are being inclusive. |
| 2.0 Help planning the promotion of PA in the school | 2.1 Social media guide | ✓ | ⭮ | ✓ | 1. **Kept.** Needed more clarity- Suggested to provide clear steps or actions to get your message across on social media. 2. **Revised.** Title changed to “Steps for planning the promotion of PA”- Too many steps. Need to account for differences between school policies. Needs to look fun. 3. **Kept.** Suggested that the list of steps are only for teachers knowledge, students only need to see the actions to be completed. |
|  | 2.2Template social media posts, posters and banners | ✓ | ⭮ | 🗶 | 1. **Kept.** Mixed responses on the usefulness of these as students do a good job by themselves. 2. **Revised.** Changed to templates for posters including suggested graphics, logos, text fonts and colors. 3. **Removed**. |
|  | 2.3 Social media planning template | ✓ | ⭮ | ✓ | 1. **Kept.** Need to break down into short periods i.e. not for the full year, 2. **Revised.** Name changed to “content planning template”- Can be used for various modes of promotion not just social media. 3. **Kept.** Some minor changes to reduce repetition and improve clarity. |
|  | 2.4Tips and tricks to improve social media presence | ✓ | ⭮ | 🔗 | 1. **Kept.** Listed suggestions that would help them. 2. **Revised.** Suggested to combined with plan for promoting PA to form new section on “brainstorming content sharing plans”- Students can come up with their own factors to consider when posting on social media e.g. how to increase engagement. 3. **Combined.** Now called ‘class brainstorming activities’ |
|  | 2.5 Plan for promoting PA | ✓ | ⭮ |  | 1. **Kept.** Resources were liked and feel they would help them plan but it needs to be simplified. 2. **Revised.** Suggested to combined with tips and tricks to form new section on “brainstorming content sharing plans”. 3. **Combined.** Now called ‘class brainstorming activities’. |
|  | 2.6 Guide to create interesting PA messages and infographics | ✓ | ⭮ | 🔗 | 1. **Kept.** Additional suggestions for the do’s and don’ts. 2. **Revised.** Combined with support for implementing the communications campaign. 3. **Combined.** Now called ‘physical activity message tools’. |
|  | 2.7 Support for implementing the communications campaign | ✓ | ⭮ |  | 1. **Kept.** May not be used as the messages they spread are usually about what is going on. 2. **Revised.** Contains guides to create interesting PA messages and infographics. Existing evidence-based message tools that can be used by schools to share their PA messages. Do’s and don’ts for sharing messages 3. **Combined.** Now called ‘physical activity message tools’. |
|  | 2.8 Approved speakers and topics covered | ✓ | ⭮ | ✓ | 1. **Kept.** Too difficult to manage as schools are spread out across the country 2. **Revised.** Name changed to “types of guest speakers”- List of different types of people and their organizations and potential talk topics. 3. **Kept.** No more changes. |
|  | 2.9 Inclusivity and diversity | ⭮ | ✓ | ✓ | 1. **Revised.** As per explanation in 1.7 above. 2. **Kept.** Need to use multiple platforms. Vary the times/ days when activities happen. Offer different levels of ability. Gather feedback before, during, and after events. 3. **Kept.** As per suggestion in 1.7 above. |
| 3.0 Help to develop communication skills | 3.1 Conduct a skills needs assessment | 🗶 | 🞣 | ✓ | 1. N/A 2. **Added.** The addition of a new “conduct a skills needs assessment” to help the teacher decide what skills to start developing first. 3. **Kept.** Class activity to allow students to discuss and assess their own skillset and areas for improvement |
|  | 3.2 Team bonding | ✓ | ⭮ | ✓ | 1. **Kept.** Ideas on how existing suggestions for team bonding could be brought to life. 2. **Revised.** Name changed to “Team working skills”- includes problem-solving and decision-making scenarios to practice 3. **Kept.** No further changes. |
|  | 3.3 Developing and practicing communication skills | ✓ | ⭮ | ✓ | 1. **Kept.** Included listening, presenting, and public speaking skills- activities and games provided on how to develop these skills 2. **Revised.** Two core skills were defined with sub-categories. Communication skills included listening, presenting and public speaking. Games were developed to help students practice these skills- Active School Flag 30-seconds and challenge cards. Team working skills included problem-solving and decision-making and developing relationships. Real-life potential scenarios were added for peer leaders to discuss and solve. 3. **Kept.** Presenting and public speaking skills should be presented in a PowerPoint with advice or tips. Additional challenges and scenarios added. |
|  | 3.4 Inclusivity and diversity | ⭮ | ✓ | ✓ | 1. **Revised.** As per explanation in 1.7 above.  2. **Kept**.  3. **Kept.** Ensure there is two-way communication to ensure the program is accessible for everyone. |
| Help to ensure inclusivity and diversity when communicating PA in the school | Importance of peer support | ⭮ | 🗶 | 🗶 | 1. **Revised.** Suggestions on how to incorporate this into active school activities. Moved so that they are integrated into each section. 2. **Removed.** Section removed and theme collapsed into the remaining thematic areas. |
|  | Offer diverse activities, emphasise that PA is for everyone |  |  |  | 1. **Revised.** Suggestions on how this could be done. Moved so that they are integrated into each section. 2. **Removed.** Section removed and theme collapsed into the remaining thematic areas. |
|  | Student voice |  |  |  | 1. **Revised.** Suggestions on how to gather and incorporate meaningful student voice. Moved so that they are integrated into each section. 2. **Removed.** Section removed and theme collapsed into the remaining thematic areas. |
| ⭮ Revised ✓ Kept 🗶 Removed 🔗 Linked 🞣 Added | | | | | |

**Additional file 6- The COMMUNICATE toolkit cover image designed by co-creators**


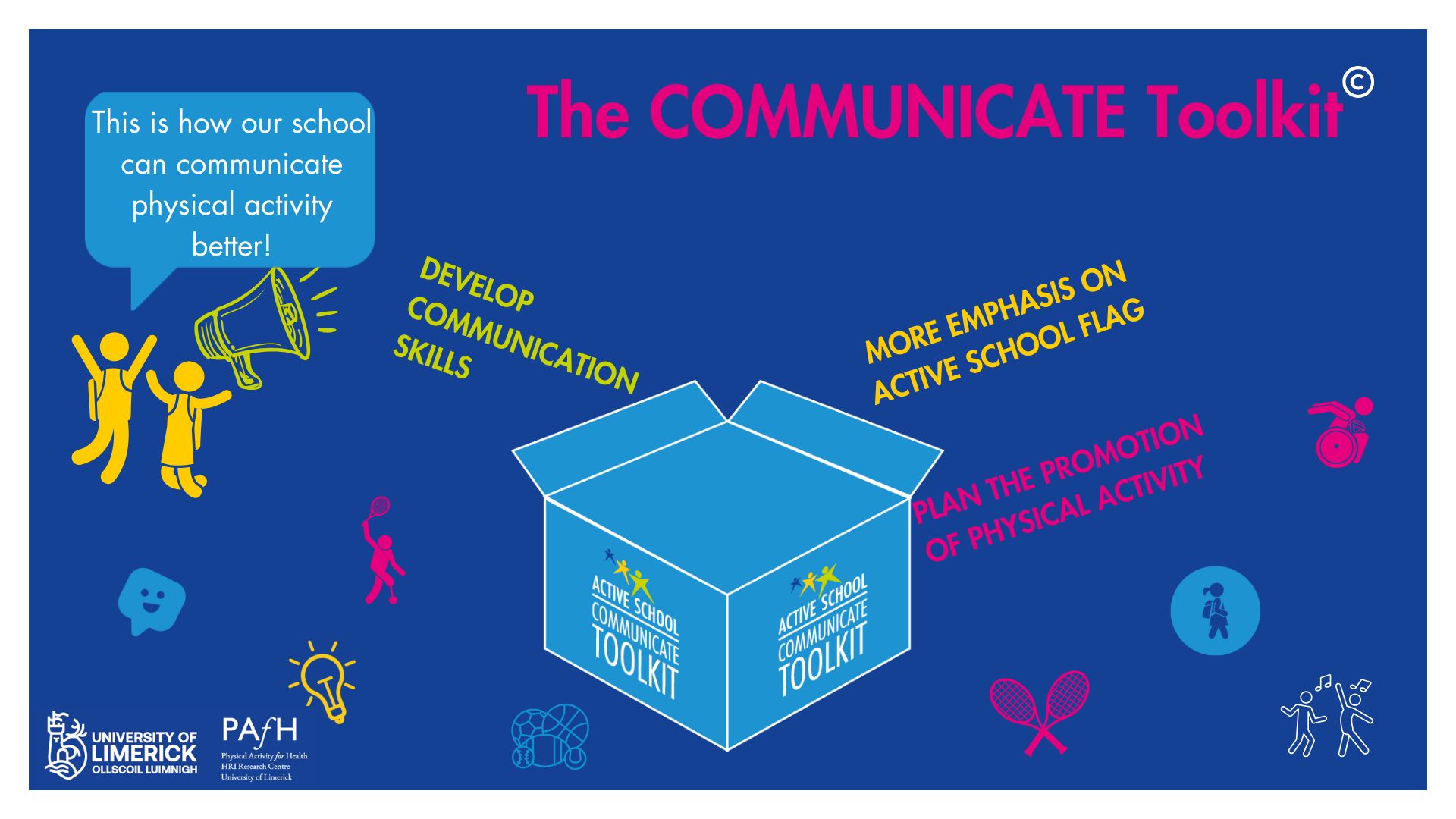


© COMMUNICATE Toolkit cover image © 2025 by [Caera Grady](https://orcid.org/my-orcid?orcid=0000-0002-9411-9066)is licensed under [CC BY-NC-SA 4.0](https://creativecommons.org/licenses/by-nc-sa/4.0/?ref=chooser-v1)
